# The Difference in Diversity between Endophytic Microorganisms in White and Grey *Zizania latifolia*

**DOI:** 10.3390/jof9111067

**Published:** 2023-11-01

**Authors:** Yipeng Li, Cailin Hu, Ruiqi Song, Zhihui Yin, Lingyun Wang, Lin Shi, Wei Li, Zhaisheng Zheng, Mengfei Yang

**Affiliations:** 1 Zhejiang Provincial Key Laboratory of Characteristic Aquatic Vegetable Breeding and Cultivation, Jinhua Academy of Agricultural Sciences, Jinhua 321000, China; lliiyyiippee@foxmail.com (Y.L.); songrq11@163.com (R.S.); lywangjh@foxmail.com (L.W.); shiyibeinan@163.com (L.S.); 2 College of Plant Protection, Hunan Agricultural University, Changsha 410128, China; cailinhu1952@163.com (C.H.); yzh15932351344@163.com (Z.Y.); liwei350551@163.com (W.L.)

**Keywords:** *Zizania latifolia*, endophyte, community, diversity, *Ustilago esculenta*

## Abstract

The *Zizania latifolia* is usually infected by the obligate parasitic fungus *Ustilago esculenta* to form an edible fleshy stem which is an aquatic vegetable called Jiaobai in China. The infection by the teliospore (T) strain of *U. esculenta* induces *Z. latifolia* forming gray fleshy stems, while the mycelia-teliospore (MT) strain of *U. esculenta* induces white fleshy stems which are more suitable for edibility than gray fleshy stems. The mechanism of this phenomenon is still largely unknown. One of the possible causes is the diversity of endophytic microbial communities between these two fleshy stems. Therefore, we utilized fungal ITS1 and bacterial 16S rDNA amplicon sequencing to investigate the diversity of endophytic microbial communities in the two different fleshy stems of *Z. latifolia.* The results revealed that the α diversity and richness of endophytic fungi in white *Z. latifolia* were significantly greater than in gray *Z. latifolia*. The dominant fungal genus in both fleshy stems was *U. esculenta*, which accounted for over 90% of the endophytic fungi. The community composition of endophytic fungi in gray and white *Z. latifolia* was different except for *U. esculenta*, and a negative correlation was observed between *U. esculenta* and other endophytic fungi. In addition, the dominant bacterial genus in gray *Z. latifolia* was *Alcaligenaceae* which is also negatively correlated with other bacterium communities. Additionally, the co-occurrence network of white *Z. latifolia* was found to have a stronger scale, connectivity, and complexity compared to that of gray *Z. latifolia*. And the detected beneficial bacteria and pathogens in the stems of *Z. latifolia* potentially compete for resources. Furthermore, the function of endophytic bacteria is more abundant than endophytic fungi in *Z. latifolia*. This research investigated the correlation between the development of *Z. latifolia* fleshy stems and endophytic microbial communities. Our findings indicate that the composition of endophytic microbial communities is closely related to the type of *Z. latifolia* fleshy stems. This research also suggests the potential utilization of specific microbial communities to enhance the growth and development of *Z. latifolia*, thereby contributing to the breeding of *Z. latifolia*.

## 1. Introduction

The *Zizania latifolia* is a perennial aquatic plant of Poaceae that grows in marshy or shallow water habitats. It is native to East Asia and widely cultivated for its edible grains and fleshy stems in China [1]. Interestingly, when the *Z. latifolia* seedlings are infected by the fungus *Ustilago esculenta*, the plants will develop edible fleshy stems called Jiaobai in China, or else the plants will not develop fleshy stems but instead edible grains. Therefore, the *Z. latifolia* is known as Chinese wild rice and the second largest aquatic vegetable after lotus (*Nelumbo nucifera*). *Z. latifolia* plays an important role in the food and economy of many rural communities in China, especially in the southern provinces of China [2,3]. Furthermore, *Z. latifolia* is of ecological importance, as it provides habitat for a variety of aquatic fauna and contributes to the overall health of wetland ecosystems [4]. The fleshy stems of *Z. latifolia* are a highly nutritious vegetable that is rich in essential amino acids, vitamins, and minerals. Its nutritional value and health benefits for humans have been widely recognized in traditional Chinese medicine for centuries [5]. For example, studies have shown that *Z. latifolia* can improve human immunity, reduce inflammation, and alleviate allergy symptoms [2]. Additionally, the high fiber content of *Z. latifolia* makes it an ideal food for promoting digestive health and preventing chronic diseases such as diabetes and cardiovascular diseases. In summary, *Z. latifolia* is a valuable and versatile plant that provides numerous health benefits and has great potential for use in functional foods and nutraceuticals [6]. The formation of the edible spindle fleshy stem of *Z. latifolia* is a unique and fascinating process that involves the interaction between the plant and the fungus *U. esculenta*. *U. esculenta* is a dimorphic fungus that belongs to the Basidiomycetes and specifically parasitizes *Z. latifolia* [7]. It infects plants through binuclear hyphae, which penetrate the stem tissue and trigger the formation of the characteristic spindle shape. As the fungus grows, it induces the plant to produce nutrients and other compounds that contribute to the nutritional value and flavor of the stem [5]. This unique symbiotic relationship between *Z. latifolia* and *U. esculenta* has been cultivated and enjoyed for centuries in China [8]. When *U. esculenta* successfully infects *Z. latifolia*, the heading and flowering of the plant are inhibited, which suppresses plant producing grains, and the stem tissue begins to expand to form a hypertrophic fleshy stem. There are two kinds of *Z. latifolia* fleshy stems: the white and tender edible fleshy stem is called white Jiaobai, while the fleshy stem full of dark teliospores is called gray Jiaobai which is not suitable for consumption as it can cause pneumonia, seriously affecting the economic value of *Z. latifolia* [9].

Accordingly, there are two types of *U. esculenta*: the T-type strain, existing in gray *Z. latifolia*, and the MT-type strain, existing in white *Z. latifolia* [7]. The MT-type *U. esculenta* has fewer surface sensors, higher species-specific ratio, fewer potential secretory proteins, and has lost some important virulence factors and host-range-related effectors [7]. The morphological characteristics and cell shape of single colonies of the two type strains are also different, and there are 146 proteins that are only expressed in the MT-type strain, while 242 proteins are only expressed in the T-type strain [10]. Additionally, the T-type strain is more adaptable to the environment and more sensitive to external signals than the MT-type strain [8]. Plants have evolved a complex defense system to fend off pathogen attacks, which involves a series of biochemical and molecular processes that ultimately leads to the activation of various defense responses [11]. In the case of *Z. latifolia*, studies have found that the enlargement of the fleshy stem is essentially a susceptible reaction of the plant to *U. esculenta* infection [12]. This process highlights the complex and dynamic interactions between plants and pathogens, which can have both positive and negative effects on plant growth and development. For example, studies have found that it can directly affect the growth of *Zizania latifolia* by changing the expression levels of plant-pathogen interaction, plant hormone signal transduction, and some metabolic pathway related genes, thereby improving the yield and quality of *Z. latifolia* [13].

Endophytes are microorganisms that live within plant tissues without causing any apparent harm to the host plan [14]. These microorganisms play a crucial role in promoting plant growth and stress resistance by enhancing nutrient absorption, competing with pathogen niches, producing antibacterial substances in the metabolic process, and inducing host plant resistance [15]. The composition of the endophytic community in plants is diversified and complicated due to the ecological environment, different parts of the host plant, and different developmental stages of the growth cycle. There have been some studies on the endophytic bacteria of *Z. latifolia*, while there is still a lack of knowledge about the endophytic fungi in *Z. latifolia* [16]. Therefore, further research is needed to explore the diversity and function of endophytic fungi in *Z. latifolia*.

In order to investigate the interaction among endophytes, *Z. latifolia,* and *U. esculenta*, we conducted ITS1 and 16S rDNA region amplicon sequencing of the microbial communities in white and gray fleshy stems of *Z. latifolia* varieties 08 and 720. The differences were analyzed in fungal diversity between white Jiaobai and gray Jiaobai to clarify the function of endophytes on the interaction between *Z. latifolia* and *U. esculenta*, which will help people utilize the specific microbial communities to enhance the growth and development of *Z. latifolia*.

## 2. Materials and Methods

### 2.1. Sample Collection

The *Z. latifolia* was planted at the experimental base of Jinhua Academy of Agricultural Sciences in Jinhua City, Zhejiang Province, China (29°01′2.83″ N, 119°37′32.09″ E). Four kinds of *Z. latifolia* were sampled, including gray Jiaobai of variety 08 (A08H), white Jiaobai of variety 08 (A08Z), gray Jiaobai of variety 7–20 (B720H), and white Jiaobai of variety 7–20 (B720Z), and from each of them was collected three replicates. The aboveground parts of the plants with the same growth status were cut for endophytes community analysis.

### 2.2. DNA Extraction and PCR Amplification

The fleshy stems of *Z. latifolia* were soaked in 70% (*v*/*v*) ethanol for 30 s. After rinsing with sterile water for three times, the surface sterilized stem tissue was cut into small cubes of 0.5 cm using a sterile surgical blade. The small cubes were then ground with mortars and pestles after pre-cooling with liquid nitrogen. Extracted total DNA from the homogenized samples was performed using the FastDNA^®^ Spin Kit for Soil (MP Biomedicals, Santa Ana, CA, USA) according to the manufacturer’s instructions. The extracted DNA was evaluated for concentration and quality using the NanoDrop^®^ ND-2000 spectrophotometer (Thermo Scientific Inc., Waltham, MA, USA) and 1% agarose gel electrophoresis and subsequently stored at −20 °C for further analysis.

The extracted DNA was used as a PCR template, and the hypervariable region V5-V7 of the bacterial 16S rDNA gene was amplified with primer pair 799F (5′-AACMGGATTAGATACCCKG-3′) and 1193R (5′-ACGTCATCCCCACCTTCC-3′) [17,18]. The ITS1 of the fungi was amplified with primer pair ITS1F (5′-CTTGGTCATTTAGAGGAAGTAA-3′) and ITS2R (5′-GCTGCGTTCTTCATCGATGC-3′) [19]. The PCR mixture includes 4 μL 5 × FastPfu buffer, 2 μL 2.5 mM dNTPs, 0.8 μL each primer (5 μM), 0.4 μL FastPfu polymerase, 10 ng of template DNA, and ddH_2_O to a final volume of 20 µL. PCR program: initial denaturation at 95 °C for 3 min, followed by 27 cycles of denaturing at 95 °C for 30 s, annealing at 55 °C for 30 s and extension at 72 °C for 45 s, and single extension at 72 °C for 10 min. The PCR product was subjected to 2% agarose gel electrophoresis and purified with the AxyPrep DNA Gel Extraction Kit (Axygen Biosciences, Union City, CA, USA) and quantified with the Quantus™ Fluorometer (Promega, Madison, WI, USA).

### 2.3. Illumina MiSeq Sequencing

Purified amplicons were pooled using the tool illumine pooling of the Illumina official website in equimolar amounts and paired-end sequenced on an Illumina MiSeq PE300 platform (Illumina, San Diego, CA, USA) according to the standard protocol from Majorbio Bio-Pharm Technology Co., Ltd. (Shanghai, China). For more info, please refer to the guidelines of Illumina MiSeq System [20].

### 2.4. Data Processing

Raw FASTQ files were de-multiplexed using an in-house perl script, and then quality-filtered by fastp version 0.19.6 [21] and merged by FLASH version 1.2.7 [22] with the following criteria:

(i) The 300 bp reads were truncated randomly receiving an average quality score of <20 over a 50 bp sliding window, and the truncated reads shorter than 50 bp or containing ambiguous characters were discarded; (ii) Only overlapped sequences longer than 10 bp were assembled according to their overlapped sequences. The maximum mismatch ratio of the overlap region is 0.2. Reads that could not be assembled were discarded; (iii) Samples were differentiated based on the barcode and primers used. The sequence direction was adjusted accordingly. The barcodes were matched exactly, while a maximum of two nucleotide mismatches were allowed in primer matching. The optimized sequences were then clustered into operational taxonomic units (OTUs) with UPARSE 7.1 [23,24] with 97% sequence similarity level. The most abundant sequence for each OTU was selected as a representative sequence. The OTU table was manually filtered, wherein chloroplast sequences in all samples were removed. To minimize the effects of sequencing depth on alpha and beta diversity measures, the number of 16S rDNA and ITS1 gene sequences from each sample were rarefied to 13,585 and 29,927, which still yielded an average Good’s coverage of 99.99% and 100%, respectively (Supplementary Table S1).

The taxonomy of each OTU representative sequence was analyzed by the RDP Classifier version 2.2 [25] against the 16S rDNA gene database (Silva138/16s_bacteria) [26] and the ITS1 gene database (https://unite.ut.ee/) [27] using a confidence threshold of 0.7. The 16S rDNA functions were predicted by PICRUSt1 (Phylogenetic Investigation of Communities by Reconstruction of Unobserved States) [28] and fungal functions were predicted by FUNGuild (Fungi Functional Guild) [29] based on OTU representative sequences.

### 2.5. Statistical Analyses

Bioinformatic analysis of the *Z. latifolia* microbiota was carried out with the Majorbio Cloud platform (https://cloud.majorbio.com, accessed on 29 December 2022). Based on the OTUs information, rarefaction curves, and alpha diversity indices including observed OTUs, Chao1 richness, Shannon index, and Good’s coverage were calculated with Mothur v1.30.1 [30]. The similarity among the microbial communities in different samples was determined by principal coordinate analysis (PCoA) based on Bray-curtis dissimilarity and principal component analysis (PCA) using the Vegan v2.5-3 package [31]. The PERMANOVA test was used to assess the percentage of variation explained by the treatment along with its statistical significance using the Vegan v2.5-3 package. The linear discriminant analysis (LDA) effect size (LEfSe) [32] (http://huttenhower.sph.harvard.edu/LEfSe, accessed on 13 May 2023) was performed to identify the significantly abundant taxa (phylum to genera) of bacteria among the different groups (LDA score > 2, *p* < 0.05). The co-occurrence networks were constructed to explore the internal community relationships across the samples [33]. A correlation between two nodes was considered to be statistically robust if the spearman’s correlation coefficient was over 0.6 or less than −0.6 and the *p*-value was less than 0.01.

## 3. Results

### 3.1. Differences in Microbial Diversity and Community Composition between White and Gray Z. latifolia

**Figure 1:**
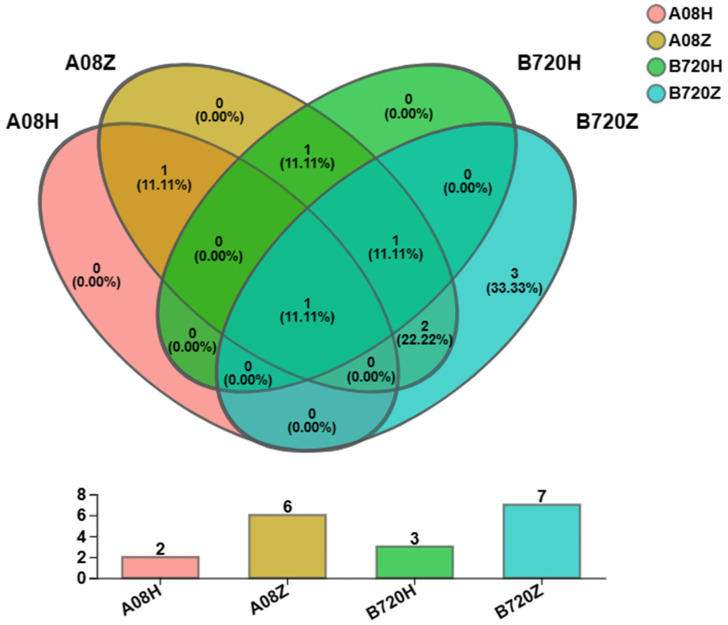
OTUs Venn analysis on endophytic fungi in *Z. latifolia*. A08H: gray *Z. latifolia* of variety 08; A08Z: white *Z. latifolia* of variety 08; B720H: gray *Z. latifolia* of variety 7–20; B720Z: white *Z. latifolia* of variety 7–20.

To examine the composition and diversity of endophytes in white and gray *Z. latifolia*, genomic DNA was extracted from four kinds of *Z. latifolia* and three replicates for each of them: gray Jiaobai of variety 08 (A08H), white Jiaobai of variety 08 (A08Z), gray Jiaobai of variety 7–20 (B720H), and white Jiaobai of variety 7–20 (B720Z). The genomic DNA was then purified and used as a PCR template to amplify fungi ITS1 and bacteria 16S rDNA. The DNA gel electrophoresis of the amplified ITS1 and 16S rDNA fragments showed distinct bands that matched the expected results in terms of size. It suggests that these fragments are appropriate for Illumina MiSeq sequencing. Operational Taxonomic Units (OTUs) are standardized markers used in phylogenetic or population genetics research for classification (Supplementary Table S2). OTUs with 97% consistency were used for clustering and species annotation of OTU sequences. The ITS1 amplicon was sequenced to obtain the fungal community classification of the twelve *Z. latifolia* samples. The fungi communities of the twelve samples were classified into one Domain, one Kingdom, two Phyla, six Classes, eight Orders, eight Families, nine Genera, nine Species, and nine OTUs. The Venn diagram indicated that the gray *Z. latifolia* A08H and B720H had two and three fungal OTUs, respectively, while the white *Z. latifolia* A08Z and B720Z had six and seven fungal OTUs. The proportion of fungal OTUs in the A08H, B720H, A08Z, and B720Z varieties to the total variety fungal OTUs was 22.22%, 33.33%, 66.66%, and 77.77%, respectively (Figure 1). Based on the results, the fungal OTUs in white *Z. latifolia* were more than that in the corresponding gray *Z. latifolia*. Furthermore, according to 16S rDNA amplicon sequencing, the endophytic bacteria OTUs in gray *Z. latifolia* A08H and B720H were as follows: one Domain, one Kingdom, three Phyla, five Classes, eight Orders, eleven Families, Fourteen Genera, fourteen Species, and fifteen OTUs. There are fourteen OTUs in both A08H and B720H (Figure S1).

**Figure 2:**
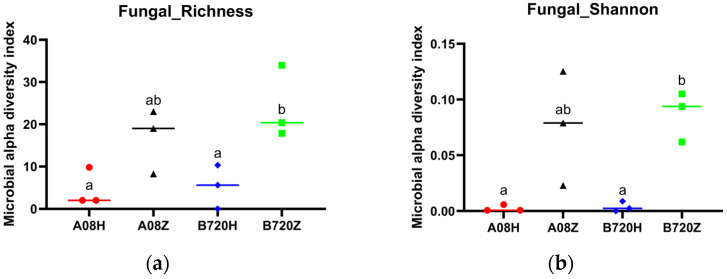
The α diversity of endophytic fungi in *Z. latifolia*. Different letters indicate significant difference at *p* < 0.05 based on Tukey’s test. (**a**) The richness of fungal community; (**b**) Diversity of fungal community. Red, black, purple and green represent the samples A08H, A08Z, B720H, B720Z, respectively.

Higher Ace index value indicates richer species, while larger Shannon index value indicates higher species diversity. The analyzed sequencing results indicate that the alpha diversity of the fungal community in gray *Z. latifolia* differs from white *Z. latifolia* (Figure 2a, Supplementary Table S3). Furthermore, the community richness and Shannon index of fungi in white *Z. latifolia* A08Z and B720Z were higher compared to gray *Z. latifolia* A08H and B720H. Additionally, the community richness and diversity of fungi were significantly higher in B720Z than in A08H and B720H (Tukey’s test, *p* < 0.05; Figure 2b). However, there was no significant difference between the gray *Z. latifolia* A08H and B720H in the richness (Figure S2a) and diversity of endophytic bacterial communities (Figure S2b). The results suggest that the composition of the fungal communities is closely related to the phenotype (i.e., gray or white fleshy stems) of *Z. latifolia* infected by *U. esculenta*.

**Figure 3:**
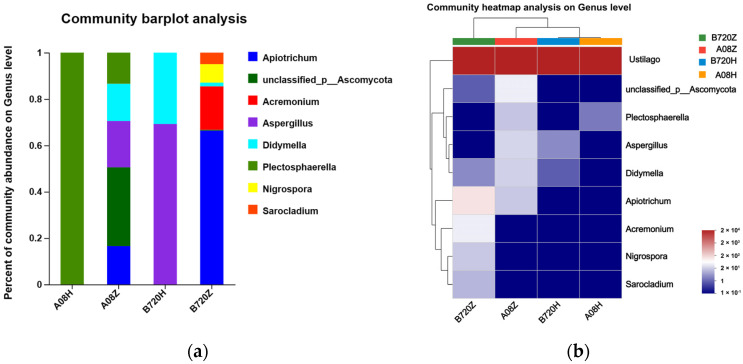
Taxonomic composition of the microbial community in *Z. latifolia.* (**a**) Relative abundance of different fungal genera excluding *U. esculenta*; (**b**) The fungal community heatmap analysis on genus level at different *Z. latifolia* varieties. The genera ranked outside the TOP10 were grouped into “Others”.

Most of the endophytic fungi in *Z. latifolia* were *U. esculenta*, but there was notable diversity among other fungi between white *Z. latifolia* and gray *Z. latifolia* (Figure 3a). The diversity of endophytic fungi in B720Z samples was more than that of B720H, the same as A08Z and A08H. B720Z samples, compared to B720H, had a higher presence of *Apiotrichum*, *Acremonium*, *Nigrospra*, and *Sarocladium*, while B720H samples were mainly composed of *Aspergillus* and *Didymella* (Figure 3a). A08Z samples, compared to A08H, had a higher presence of *Ascomycota*, *Aspergillus*, *Didymella*, and *Apiotrichum*, while A08H predominantly contained *Plectosphaerella* (Figure 3b). The endophytic fungi communities show a bigger difference between A08Z and B720Z compared to the difference between A08H and B720H.

**Figure 4:**
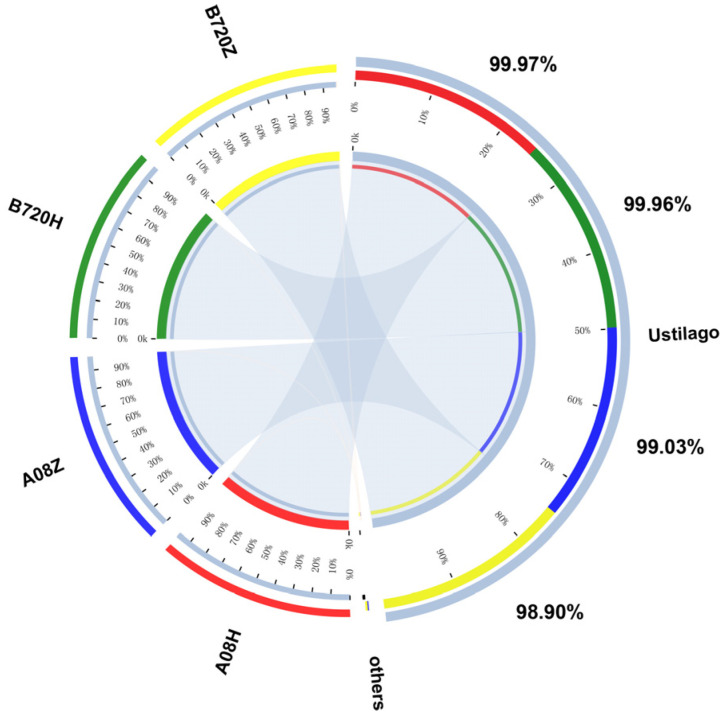
The relationship between *Z. latifolia* varieties and their endophytic fungi species. Different colors represent different varieties of *Z. latifolia*, and the numbers on the right part of the diagram represent the proportion of *U. esculenta* in endophytic fungi in different varieties of *Z. latifolia*.

Further analysis of the relationship between the *Z. latifolia* varieties and endophytic fungi species was performed with Circos software Circos-0.67-7 which revealed that *U. esculenta* accounted for 98.90%, 99.96%, 99.03%, and 99.97% of the fungi present in B720Z, B720H, A08Z, and A08H, respectively (Figure 4).

What is more, the unclassified *Alcaligenaceae* was the dominant endophytic bacteria in both gray *Z. latifolia* A08H and B720H accounted for 97% and 90%, respectively (Figure S3a). However, the proportion of other endophytic bacteria species present in A08H and B720H were different. B720H had higher percentages of *Sphingomonas* and *Rhodobacter* than that in A08H (Figure S3b). The endophytic bacteria found in A08H consisted of 97% unclassified *Alcaligenaceae*, 2.2% *Delftia*, 0.67% *Sphingomonas*, 0.025% *Rhodobacter*, and 0.66% other genera (Figure S4). However, B720H had dominant endophytic bacteria mainly from unclassified *Alcaligenaceae* (90%) and *Sphingomonas* (4.8%).

**Figure 5:**
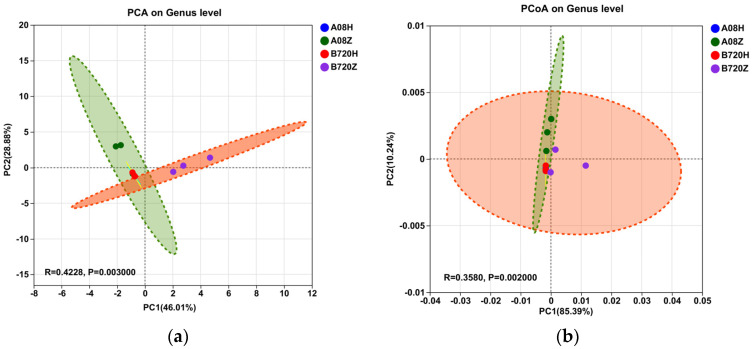
Distribution patterns of endophytic fungal communities in *Z. latifolia* varieties. Principal co-ordinate analysis revealed the beta diversities of fungi based on the full dataset. The significance tests of compartment niche effects and different *Z. latifolia* varieties on the microbial community structure were performed with the analysis of similarity (ANOSIM) based on Bray-Curtis dissimilarities among samples. The ellipses represent 0.80 of confidence intervals of each treatment. (**a**) Principal component analysis of fungal communities between different compartment niches of different *Z. latifolia* varieties; (**b**) Principal axis analysis of fungal communities between different compartment niches of different *Z. latifolia* varieties.

PCA similarity analysis revealed that the fungal communities in *Z. latifolia* varieties formed two clusters. The fungal community composition in B720Z significantly differed from that of other samples (ANOSIM: R = 0.4043, *p* < 0.05; Figure 5a). The PCA similarity analysis, based on Bray-Curtis distance, revealed that the true community composition in A08Z was also significantly different from other samples (ANOSIM: R = 0.3781, *p*< 0.05; Figure 5b). However, the community composition of endophytic bacteria was similar between the gray *Z. latifolia* A08H and B720H (Figure S5), which is consistent with the previous analysis of community composition.

### 3.2. Fungal Abundance Affects Endophytic Fungi Community Composition in Z. latifolia

**Figure 6:**
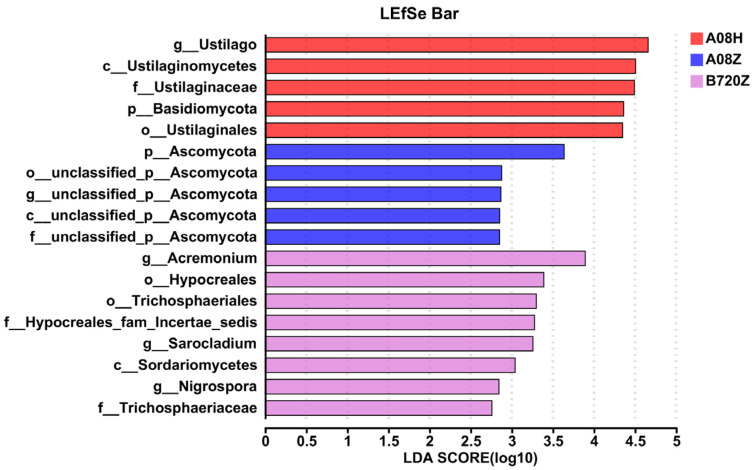
Fungal biomarkers in different *Z. latifolia* varieties based on linear discriminant analysis (LDA). The LDA showed the different abundance of endophytic bacterial genera in white *Z. latifolia* and gray *Z. latifolia*. The significance was determined using the Kruskal-Wallis test with *p* < 0.05 and logarithmic LDA score > 2.0, wherein the B720H was out of this range. Genera with a relative abundance of less than 0.1% were not included. Different colors represent different varieties of *Z. latifolia*.

According to LEfSe linear discriminant analysis (LDA), we examined the impact of fungal abundance to fungal community differences between white and gray *Z. latifolia*. The results revealed that white and gray *Z. latifolia* showed variation on the number and species of endophytic fungi. *Ustilago* was enriched in A08H and had the most impact on the fungal community composition. On the other hand, *Ascomycota* and unidentified Ascomycota species were enriched in A08Z. The fungal community in B720Z was found to be diverse, and the *Acremonium* was mainly enriched and affected the endophytic fungi species (Figure 6). The species difference was most affected by the main enriched unclassified *Alcaligenaceae* in A08H, while in B720H, the most impact on species difference was *Stenotrophomonas* (Figure S6).

### 3.3. Analysis of Microbial Network Topology in Z. latifolia

**Figure 7:**
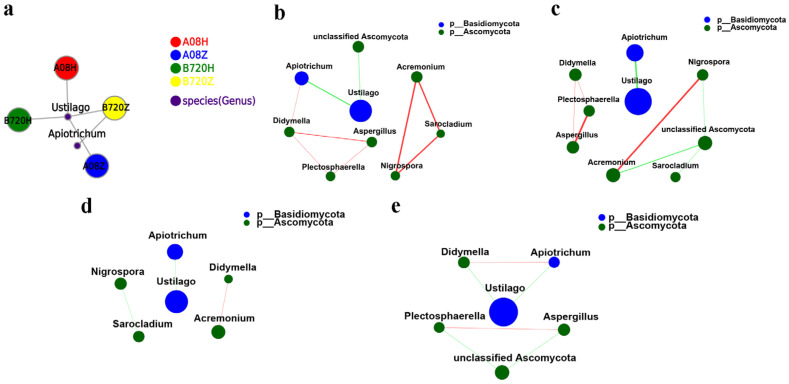
Co-occurrence network diagram of endophytic fungi in different *Z. latifolia* varieties. Nodes represent *Z. latifolia* samples or endophytic fungi species, and the connection between *Z. latifolia* sample node and endophytic fungi species node represents that the *Z. latifolia* sample contains the endophytic fungi species (genus level). The results show species with abundance greater than 50 (**a**), the microbial co-occurrence network patterns of all *Z. latifolia* samples (**b**), the microbial co-occurrence network patterns of white *Z. latifolia* (**c**), the microbial co-occurrence network patterns of white *Z. latifolia* B720Z (**d**), the microbial co-occurrence network patterns of white *Z. latifolia* A08Z (**e**). Node size is proportional to the degree of connection. The red lines indicate positive correlation and the green lines indicate negative correlation. The thickness of the lines indicates the correlation coefficient. The thicker the line, the stronger the correlation between fungi species. The more numerous the lines, the closer the relationship between the fungi species and other species. The significance was determined using the Spearman test with *p* < 0.05.

The distribution of dominant genera (with an average relative abundance of over 0.1%) varied among the different *Z. latifolia* samples (Figure 7). The fungal community network in the four kinds of samples was relatively simple. As mentioned above, *U. esculenta* was abundantly present in both white and gray *Z. latifolia*, while the *Apictrichum* uniquely appeared in B720Z (Figure 7a). We analyzed the correlation between dominant fungus *U. esculenta* and other fungi in all samples using single factor correlation network analysis (Figure 7a–e). The results showed a negative interaction between *U. esculenta* and *Apiotrichum*, which was stronger than the negative interaction between *U. esculenta* and unclassified *Ascomycota*. On the other hand, there was a close positive interaction among *Sarocladium*, *Nigrospora*, and *Acremonium* (Figure 7b). The correlation among fungi in white *Z. latifolia* showed that the dominant fungus *U. esculenta* had only negative interaction with the other endophytic fungi, and the *Apiotrichum* was negatively correlated with *U. esculenta*. (Figure 7c). Further analysis of the relationship among fungi in B720Z revealed similar results, wherein the dominant fungi *U. esculenta* had only negative interaction with the other endophytic fungi (Figure 7d,e). B720H had more endophytic fungi species than A08H, for example, B720H had *Romboutsia*, *Xanthomonas*, and *Escherichia-Shigella* which were not found in the top 50 of species abundance in A08H. However, the *Delftia*, *Sphingomonas,* and unclassified *Alcaligenaceae* were present in both B720H and A08H (Figure S7a). Furthermore, the analysis showed negative correlation between the dominant bacterium unclassified *Alcaligenaceae* and the other endophytic bacteria in gray *Z. latifolia*, as indicated by the single factor analysis of the network correlation (Figure S7b). The analysis of the network relationship between species in A08H and B720H showed that the endophytic bacterial communities in A08H displayed closer relationships than those in B720H. Interestingly, the dominant endophytic bacterium unclassified *Alcaligenaceae* in A08H had negative correlation with the other endophytic bacteria (Figure S7c), while in B720H, it had positive interaction with *Thauera* (Figure S7d).

**Figure 8:**
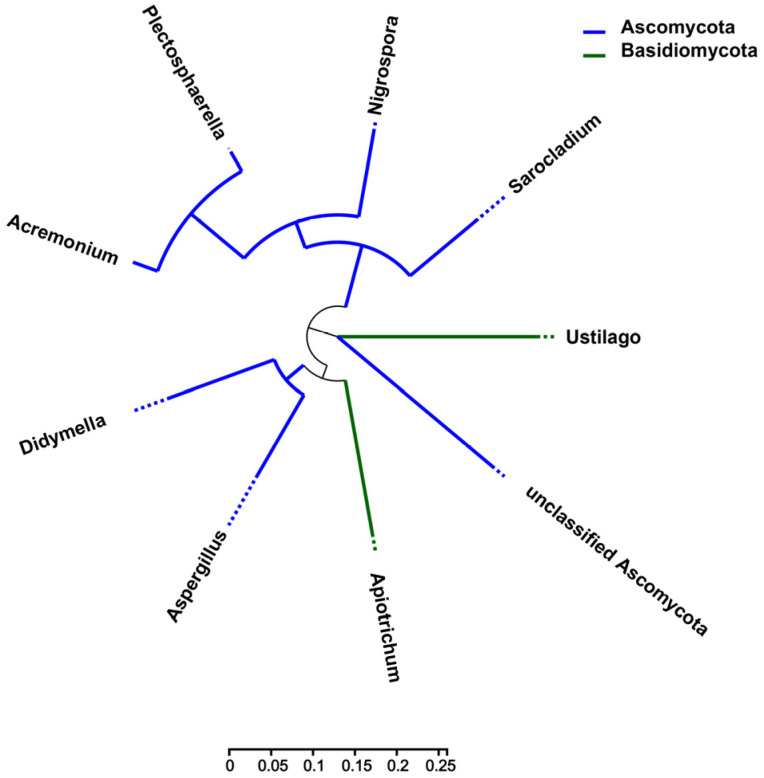
Phylogenetic tree of endophytic fungi in white and gray *Z. latifolia* varieties. Each branch of the phylogenetic tree represents a class of endophytic fungi species. The branch length represents the evolutionary distance between the two fungal species.

To analyze the evolutionary relationship of microbial communities in *Z. latifolia*, a phylogenetic tree of endophytic fungi and bacteria was constructed using FastTree. The results indicated that the endophytic fungi were categorized into three primary branches: *Didymella*, *Aspergillus*, and *Apiotrichum* were in one branch, *Acremonium*, *Plectosphaerella*, *Nigrospora*, and *Sarocladium* were in one branch, and *U. esculenta* and unclassified *Ascomycota* formed the third branch, which indicates the close evolutionary relationship between *Ascomycota* and *U. esculenta* (Figure 8). The endophytic bacteria were also divided into three branches. Two branches belong to *Proteobacteria*, among them, the dominant bacterium *Alcaligenaceae* had the closest evolutionary relationship with *Herbaspirillum* (Figure S8). Additionally, *Bacillus* and *Romboutsia* of Firmicutes had close evolutionary relationships with *Leifsonia* (Figure S8).

### 3.4. Functional Analysis of Microorganisms in Z. latifolia

**Figure 9:**
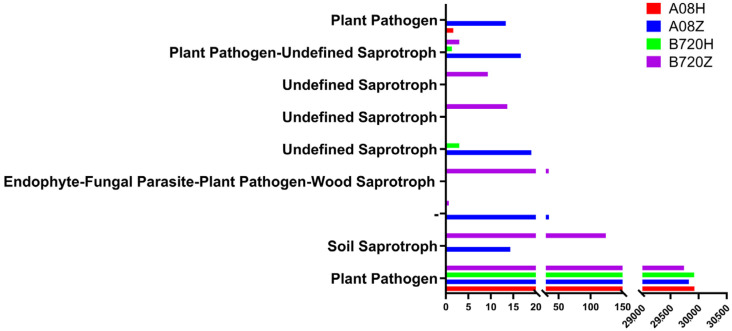
Functional prediction of endophytic fungi in *Z. latifolia* varieties. The ordinate is the functional classification of endophytic fungi in different *Z. latifolia* varieties, and the abscissa is the abundance of each functional classification in different samples.

In this study, FUNGuild software 1.0 was utilized to cluster the functions of fungi in *Z. latifolia*. The results indicated that the fungi present in all *Z. latifolia* varieties were primarily plant pathogens. Endophytic fungi in white *Z. latifolia* exhibit a wider range of functions compared to those found in gray *Z. latifolia*, and the endophytic fungi in white *Z. latifolia* have functions that include undefined saprotroph, wood saprotroph, and soil saprotroph (Figure 9). Kyoto Encyclopedia of Genes and Genomes (KEGG) is a large knowledgebase for systematically analyzing gene functions and linking genomic information and functional information. We used KEGG to predict the functions of endophytic bacteria in gray *Z. latifolia*. The functions of endophytic bacteria in B720H and A08H were mainly enriched in global and overview maps, amino acid metabolism, carbohydrate metabolism, membrane transduction, and cellular community-prokaryotes (Figure S9).

## 4. Discussion

The composition of plant endophytic microorganism communities significantly contributes to plant health [34,35]. This study revealed that white *Z. latifolia* had higher diversity and richness of endophytic fungi compared to gray *Z. latifolia*. The reason could be the damage caused by the strong infection ability of T-type *U. esculenta*, which led to the destruction of the original fungi community structure in *Z. latifolia* stems. Additionally, the short incubation period of T-type *U. esculenta* in *Z. latifolia* and the quick formation of chlamydospore piles in swollen fleshy stems could also contribute to this phenomenon. The strong infection capability of T-type *U. esculenta* leads it to form a large number of chlamydospores to occupy living space, making it difficult for other endophytic fungi to coexist in the swollen fleshy stems of *Z. latifolia*. However, the MT-type strains have a longer latent period in *Z. latifolia*, and the spore piles form in the late stages of fleshy stem development [36]. The late formation of spore piles produced by MT type *U. esculenta* provides available living space for other endophytic fungi.

This study found that there are differences in the types of endophytic fungi between white and gray *Z. latifolia*. The white *Z. latifolia* B720Z had a higher abundance of endophytic *Apiotrichum*, *Acremonium*, and *Nigrospra* compared to A08Z. Additionally, the main endophytic fungi, except *U. esculenta*, identified in B720H were *Aspergillus* and *Didymella*, while *Plectosphaerella* dominated in A08H. There was no significant difference in the diversity of endophytic bacteria between the two gray *Z. latifolia* varieties, but the proportion of bacterial species was different. These findings suggest that the infection of *U. esculenta* affected the microbial community structure of *Z. latifolia* stems, which was different between white and gray *Z. latifolia*. The function of endophytic fungi is closely related to the growth of host plants [37]. For instance, *Acremonium* sp., an endophytic fungus found in rice seeds in Sri Lanka, has the potential to enhance rice growth [38]. It also occurs in *Z. latifolia* and may have potential benefits for *Z. latifolia* stem growth. *U. esculenta* stimulates stem swelling under suitable environmental conditions [12].

When certain fungi infect a host plant, they stimulate the host tissue to expand by increasing the size and quantity of cells, resulting in the formation of swollen tissue. Conversely, infection by *U. esculenta* hinders the growth of inflorescences and the production of seeds, redirecting the energy required for these processes towards the development of the fleshy stem [39].

It was discovered that the metabolites produced by *Nigrospora* sp., an endophytic fungus in *Aconitum carmichaeli*, exhibit significant antiviral activity against influenza virus H1N1 [40]. However, the abundance and variety of this beneficial fungus are decreased in *Z. latifolia* due to the strong infection ability of *U. esculenta* and the formation of teliospores. Previous studies have shown that the symbiosis of *U. esculenta* and *Z. latifolia* not only produces edible fleshy stems, but also improves the photosynthesis and growth of *Z. latifolia* [41]. This may be related to the function of carbohydrate metabolism of endophytic bacteria in *Z. latifolia* predicted in this research, but its specific mechanism needs to be further verified. And it is still unclear whether the decrease of this bacterium will affect the growth of the other bacterium on the virus in *Z. latifolia*. This study proposed a new approach to cultivate white *Z. latifolia* by optimizing the diversity and richness of endophytic fungi, which aims to delay or inhibit the formation of teliospores of T-type *U. esculenta*, and ultimately increase the yield of white *Z. latifolia*.

## 5. Conclusions

This study explored the diversity and relationship of endophytic microbial communities in white *Z. latifolia* and gray *Z. latifolia*. Both white *Z. latifolia* and gray *Z. latifolia* have a high abundance of the same dominant fungus *U. esculenta* and dominant bacterium unclassified *Alcaligenaceae*, as well as other unique dominant microorganisms. Except for *U. esculenta*, the abundance of the other fungi in white *Z. latifolia* and gray *Z. latifolia* was different. Similarly, except for *Alcaligenaceae*, the abundance of other endophytic bacteria was different between white *Z. latifolia* and gray *Z. latifolia*. Furthermore, this study found that white *Z. latifolia* had a higher diversity and richness of endophytic fungi compared to gray *Z. latifolia*. Additionally, except for *U. esculenta*, the richness of the other endophytic fungi varied between the two varieties of white *Z. latifolia*. However, there was no significant difference in the richness and diversity of endophytic bacteria between the two varieties of white *Z. latifolia*. The endophytic fungal network in white *Z. latifolia* is larger and more complex compared to gray *Z. latifolia*, which is attributed to the stronger infection ability and quicker formation of chlamydospore piles of *U. esculenta* in gray *Z. latifolia* to suppress other endophytes for growth and development. Moreover, our findings indicate that *Z. latifolia* forming white or gray stems is linked to endophytic microorganism composition.

## Data Availability

The raw sequence files were uploaded to the NCBI Sequence Read Archive and the accession number is PRJNA1028164.

## Supplementary Materials

The following supporting information can be downloaded at: https://www.mdpi.com/article/10.3390/jof9111067/s1.
