# Supplementary material for: The Difference in Diversity between Endophytic Microorganisms in White and Grey Zizania latifolia
Source: J Fungi (Basel). 2023 Nov 1;9(11):1067. doi: 10.3390/jof9111067 (PMC10672487; doi:10.3390/jof9111067)
Supplement: Supplementary file 1 [file jof-09-01067-s001.zip › Supplementary Figures.pdf]

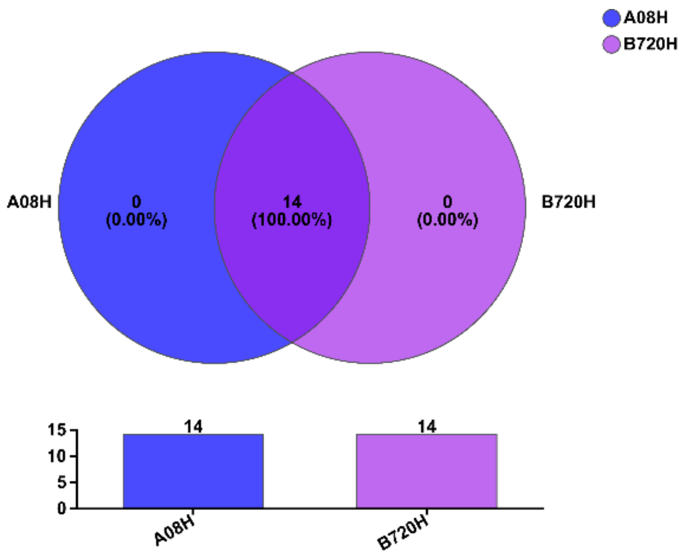

Figure S1. OTUs Venn analysis on endophytic bacteria in different gray *Z. latifolia* varieties.

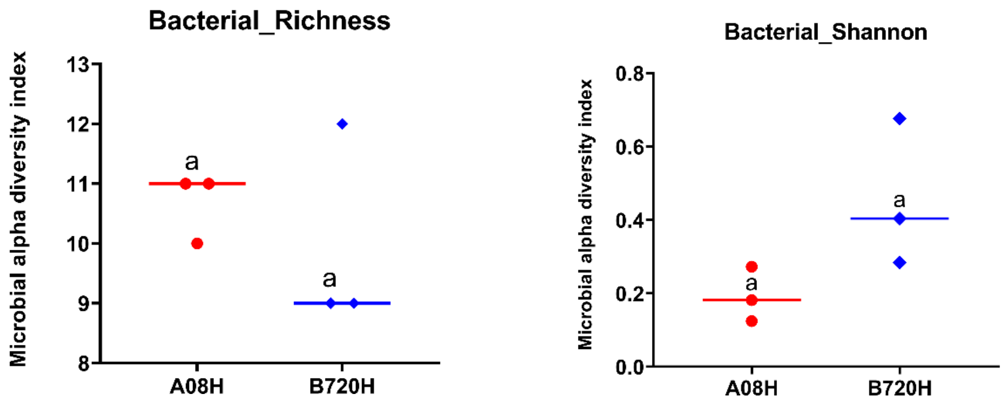

(**a**) (**b**)

Figure S2. The α diversity of endophytic bacteria in gray *Z. latifolia* varieties. There is no significant difference between two varieties at $p < 0.05$ based on Tukey's test. (**a**) The richness of bacterial community; (**b**) Diversity of bacterial community. Red and purple represent the samples A08H, B720H, respectively.

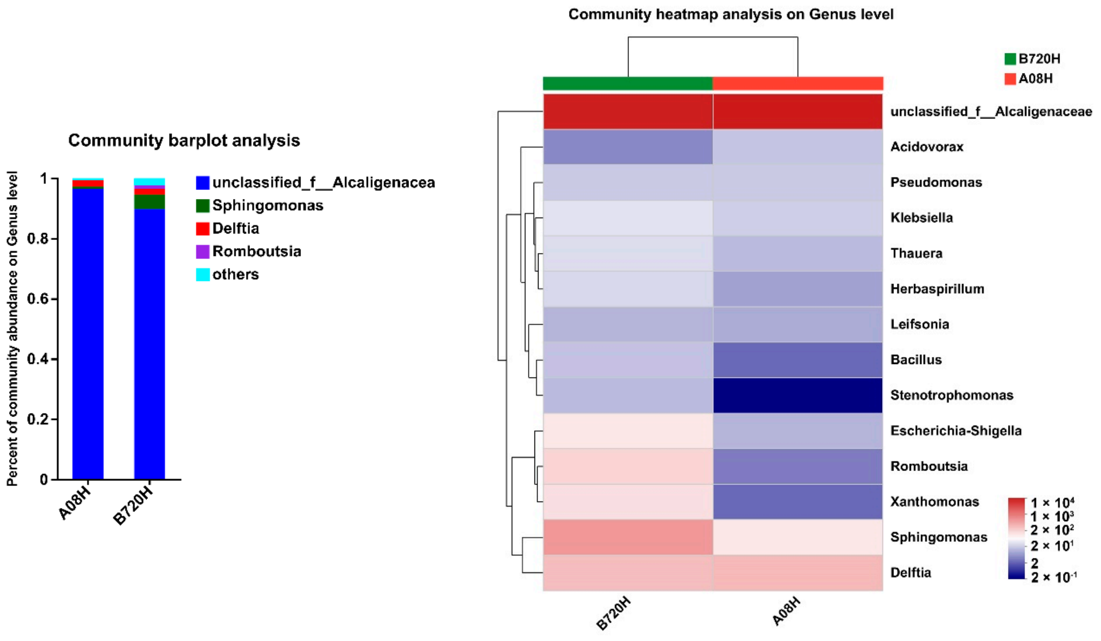

(a) (b)

Figure S3. Taxonomic composition of the microbial community in gray *Z. latifolia* varieties. Relative abundance of different bacteria genus. The genus ranked outside the TOP10 were grouped into "Others". (**a**) Relative abundance of different bacterial genus; (**b**) The bacterial community heatmap analysis on genus level at different *Z. latifolia* varieties.

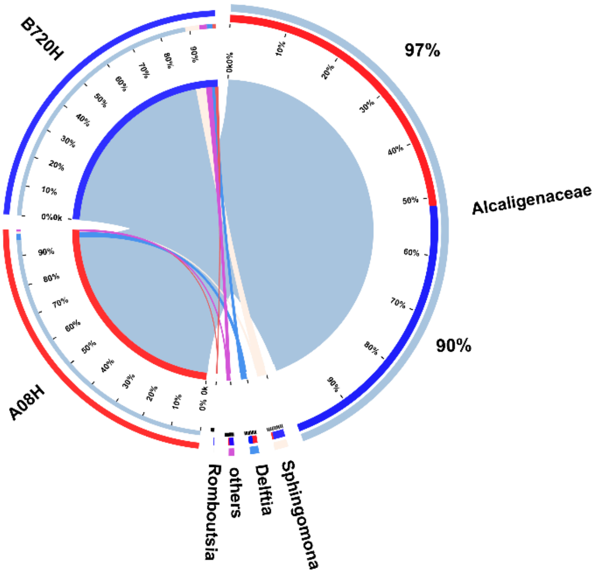

Figure S4. The relationship between gray *Z. latifolia* varieties and endophytic bacteria species. Different colors represent different varieties of *Z. latifolia*, and the numbers on right part of the diagram represent the proportion of unclassified *Alcaligenaceae* in endophytic bacteria in different varieties of *Z. latifolia*.

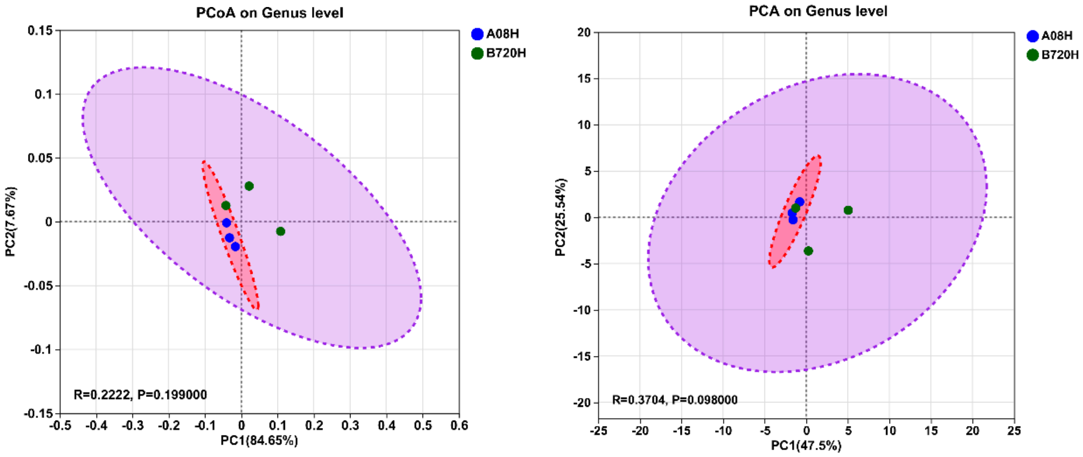

(a) (b)

Figure S5. Distribution patterns of endophytic bacterial communities in gray *Z. latifolia* varieties. The significance tests of compartment niches effects and different *Z. latifolia* varieties on the microbial community structure were performed using the analysis of similarity (ANOSIM) based on Bray-Curtis dissimilarities among samples. The ellipses represent 0.80 of confidence intervals of each treatment. (a) Principal component analysis of fungal communities between different compartment niches of different *Z. latifolia* varieties. (b) Principal axis analysis of fungal communities between different compartment niches of different *Z. latifolia* varieties.

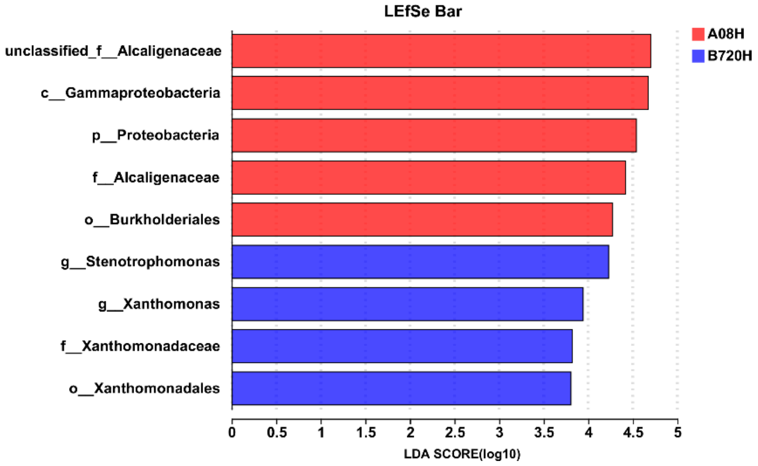

Figure S6. Bacterial biomarkers in different gray *Z. latifolia* varieties based on linear discriminant analysis (LDA). The LDA showed different abundance of endophytic bacterial genera in gray *Z. latifolia* varieties. The significance was determined using the Kruskal-Wallis test with $p < 0.05$ and logarithmic LDA score > 2.0. Bacterial genera with a relative abundance of less than 0.1% are not included.

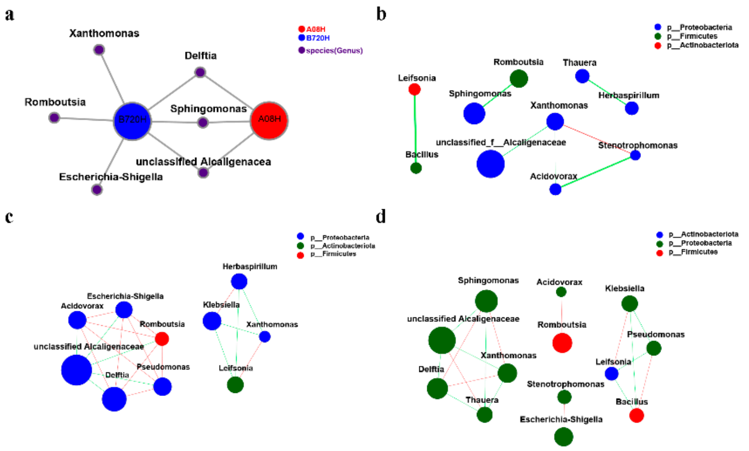

Figure S7. Co-occurrence network diagram of endophytic bacteria in gray *Z. latifolia* varieties. Nodes represent *Z. latifolia* samples or endophytic bacteria species, and the connection between *Z. latifolia* sample node and endophytic bacteria species node represents that the *Z. latifolia* sample contains the bacteria species (genus level). The results show species with abundance greater than 50 (a), the microbial co-occurrence network patterns of all *Z. latifolia* samples (b), the microbial co-occurrence network patterns of gray *Z. latifolia* B720H (c), the microbial co-occurrence network patterns of gray *Z. latifolia* A08H (d). Node size is proportional to the degree of connection. The red lines indicated positive correlation, and green lines indicated negative correlation. The thickness of the lines indicated the correlation coefficient. The thicker line, the stronger correlation between bacteria species. The more lines, the closer relationship between the bacterial species and other species. The significance was determined using the Spearman test with $p < 0.05$.

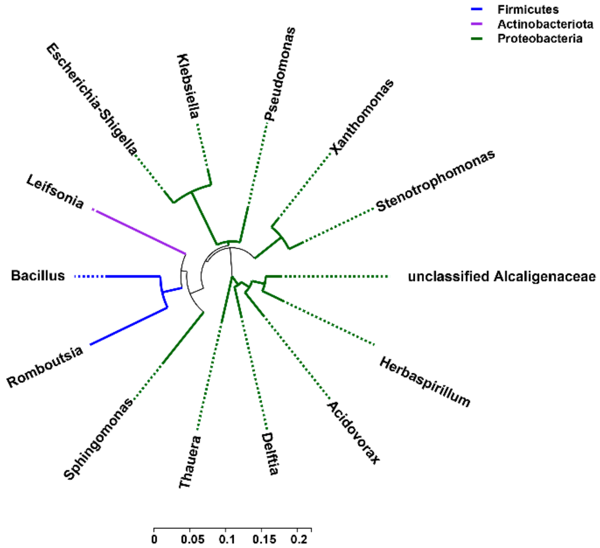

Figure S8. Phylogenetic tree of endophytic bacteria in gray *Z. latifolia* varieties. Each branch of the phylogenetic tree represents a class of endophytic bacteria species. The branch length represents the evolutionary distance between the two bacterial species.

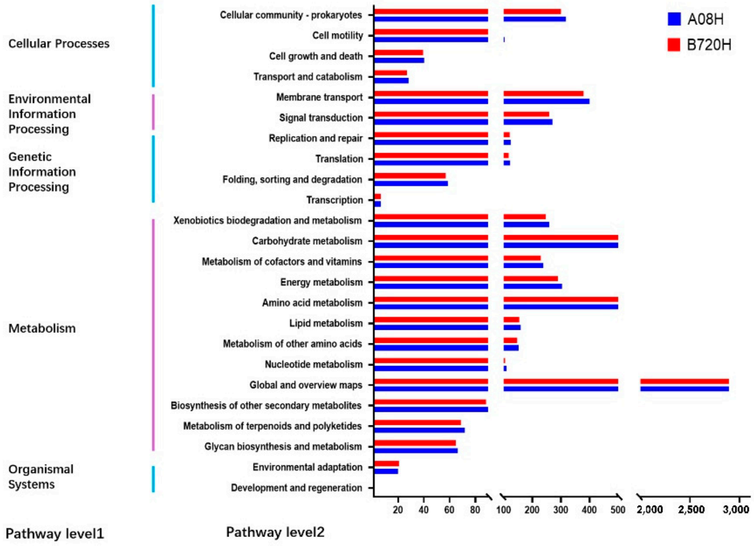

Figure S9. KEGG Functional prediction of endophytic bacteria in gray *Z. latifolia* varieties. The functional clustering of endophytic bacteria in gray *Z. latifolia* on level 1 and level 2 of KEGG Pathway, and the ordinate is the functional classification of endophytic bacteria in different *Z. latifolia* varieties, the abscissa is the abundance of each functional classification in different samples.
